# Discovery and Characterization of a Potent Interleukin-6 Binding Peptide with Neutralizing Activity *In Vivo*

**DOI:** 10.1371/journal.pone.0141330

**Published:** 2015-11-10

**Authors:** Sheila Ranganath, Ashok Bhandari, Nicole Avitahl-Curtis, Jaimee McMahon, Derek Wachtel, Jenny Zhang, Christopher Leitheiser, Sylvie G. Bernier, Guang Liu, Tran T. Tran, Herodion Celino, Jenny Tobin, Joon Jung, Hong Zhao, Katie E. Glen, Chris Graul, Aliesha Griffin, Wayne C. Schairer, Carolyn Higgins, Tammi L. Reza, Eva Mowe, Sam Rivers, Sonya Scott, Alex Monreal, Courtney Shea, Greg Bourne, Casey Coons, Adaline Smith, Kim Tang, Ramya A. Mandyam, Jaime Masferrer, David Liu, Dinesh V. Patel, Angelika Fretzen, Craig A. Murphy, G. Todd Milne, Mark L. Smythe, Kenneth E. Carlson

**Affiliations:** 1 Discovery Biology, Ironwood Pharmaceuticals, Cambridge, MA, United States of America; 2 Chemistry, Protagonist Therapeutics, Milpitas, CA, United States of America; 3 Discovery Pharmacology, Ironwood Pharmaceuticals, Cambridge, MA, United States of America; 4 Institute for Molecular Biosciences, The University of Queensland, Brisbane, Australia; 5 DMPK, Ironwood Pharmaceuticals, Cambridge, MA, United States of America; 6 Pharmaceutical Development, Ironwood Pharmaceuticals, Cambridge, MA, United States of America; 7 Protagonist Pty Ltd, Therapeutics Pty Ltd, Brisbane, Australia; 8 Chemistry, Ironwood Pharmaceuticals, Cambridge, MA, United States of America; 9 Discovery Toxicology, Ironwood Pharmaceuticals, Cambridge, MA, United States of America; 10 Biology, Protagonist Therapeutics, Milpitas, CA, United States of America; Tecnologico de Monterrey, MEXICO

## Abstract

Interleukin-6 (IL-6) is an important member of the cytokine superfamily, exerting pleiotropic actions on many physiological processes. Over-production of IL-6 is a hallmark of immune-mediated inflammatory diseases such as Castleman’s Disease (CD) and rheumatoid arthritis (RA). Antagonism of the interleukin IL-6/IL-6 receptor (IL-6R)/gp130 signaling complex continues to show promise as a therapeutic target. Monoclonal antibodies (mAbs) directed against components of this complex have been approved as therapeutics for both CD and RA. To potentially provide an additional modality to antagonize IL-6 induced pathophysiology, a peptide-based antagonist approach was undertaken. Using a combination of molecular design, phage-display, and medicinal chemistry, disulfide-rich peptides (DRPs) directed against IL-6 were developed with low nanomolar potency in inhibiting IL-6-induced pSTAT3 in U937 monocytic cells. Targeted PEGylation of IL-6 binding peptides resulted in molecules that retained their potency against IL-6 and had a prolongation of their pharmacokinetic (PK) profiles in rodents and monkeys. One such peptide, PN-2921, contained a 40 kDa polyethylene glycol (PEG) moiety and inhibited IL-6-induced pSTAT3 in U937 cells with sub-nM potency and possessed 23, 36, and 59 h PK half-life values in mice, rats, and cynomolgus monkeys, respectively. Parenteral administration of PN-2921 to mice and cynomolgus monkeys potently inhibited IL-6-induced biomarker responses, with significant reductions in the acute inflammatory phase proteins, serum amyloid A (SAA) and C-reactive protein (CRP). This potent, PEGylated IL-6 binding peptide offers a new approach to antagonize IL-6-induced signaling and associated pathophysiology.

## Introduction

IL-6 is an important member of the cytokine superfamily, known to exert pleiotropic actions on multiple physiological processes including immune, metabolic and neoplastic functions [1]. Originally identified as a potent stimulator of B cell differentiation and function, IL-6 also plays key roles in the differentiation of cytotoxic T cell and T helper cells [2,3]. During the inflammatory response, over-production of IL-6 results in fever, fatigue, loss of appetite and rapid production of the acute phase response proteins, serum amyloid A (SAA) and C-reactive protein (CRP). Over-production of IL-6 is a hallmark of many immune-mediated inflammatory diseases and is known to significantly contribute to the pathophysiology of CD and RA [4].

IL-6 is a four helix protein containing 184 amino acids that binds to the soluble and membrane-bound forms of the IL-6R with nanomolar affinity [5]. Upon binding, the IL-6/IL-6R complex associates with the ubiquitous protein gp130 and induces its dimerization and subsequent activation of the JAK/STAT pathway, resulting in the phosphorylation of STAT3 and other signaling proteins [6].

Antagonism of IL-6-induced signaling is of great therapeutic interest. Two antibody-based therapeutics targeting components of this signaling complex have been approved recently; a monoclonal antibody directed against the IL-6 receptor, tociluzimab, approved for the treatment of CD, RA and juvenile idiopathic arthritis [7], and a monoclonal antibody (mAb) specific for IL-6, siltuximab, approved for the treatment of CD [8]. Several other biologics, targeting either the cytokine or the receptor, are in development for these and other indications.

Biologic approaches to antagonize the IL-6/IL-6R complex have resulted in two successful therapeutics to date, but the critical importance of IL-6 in human pathophysiology warrants investigation of additional therapeutic modalities. Small molecule approaches have traditionally not been successful in blocking cytokine interactions. An epoxide-containing small molecule, TB-2-081, was shown to inhibit IL-6-induced proliferation of TF-1 cells and to reverse pancreatitis-induced pain in rats [9], but it is unlikely that a molecule containing a reactive pharmacophore would be a safe therapeutic. A small protein approach based on avimers^TM^ was undertaken by Avidia and generated C236 (AMG-220), an 18 kDa protein that bound to IL-6 with sub-nanomolar affinity and prolonged PK [10]. This molecule entered clinical development in 2007 but no further progress has been reported [11].

To date, peptide-based approaches disrupting cytokine/cytokine receptor interactions have had limited success, likely due to the biophysical and stability-based limitations conferred by unstructured peptides. One such example is the identification of Guess 4a, a 7 amino acid linear peptide antagonist of murine IL-6 [12]. Guess 4a was effective in blocking IL-6-mediated growth in 7TD1 cells, but its weak potency (30 μM) and linear structure (making it susceptible to rapid biological or chemical degradation) make it unsuitable as an effective therapeutic. Peptide-based therapeutics have been successfully developed against other molecular targets, as exemplified by the approval of pasireotide (Signifor) for the treatment of Cushing’s disease [13] and the disulfide-rich linaclotide (LINZESS®) for the treatment of irritable bowel syndrome with constipation (IBS-C) and idiopathic and chronic constipation [14]. These examples highlight the advantages of using a constrained peptide approach to increase biological and chemical stability compared to unstructured peptides [15]. This paper describes the successful application of molecular design, phage display and medicinal chemistry techniques to identify a DRP that potently inhibits IL-6 activity both in vitro and in vivo.

## Materials and Methods

### Molecular design

**Fig 1:**
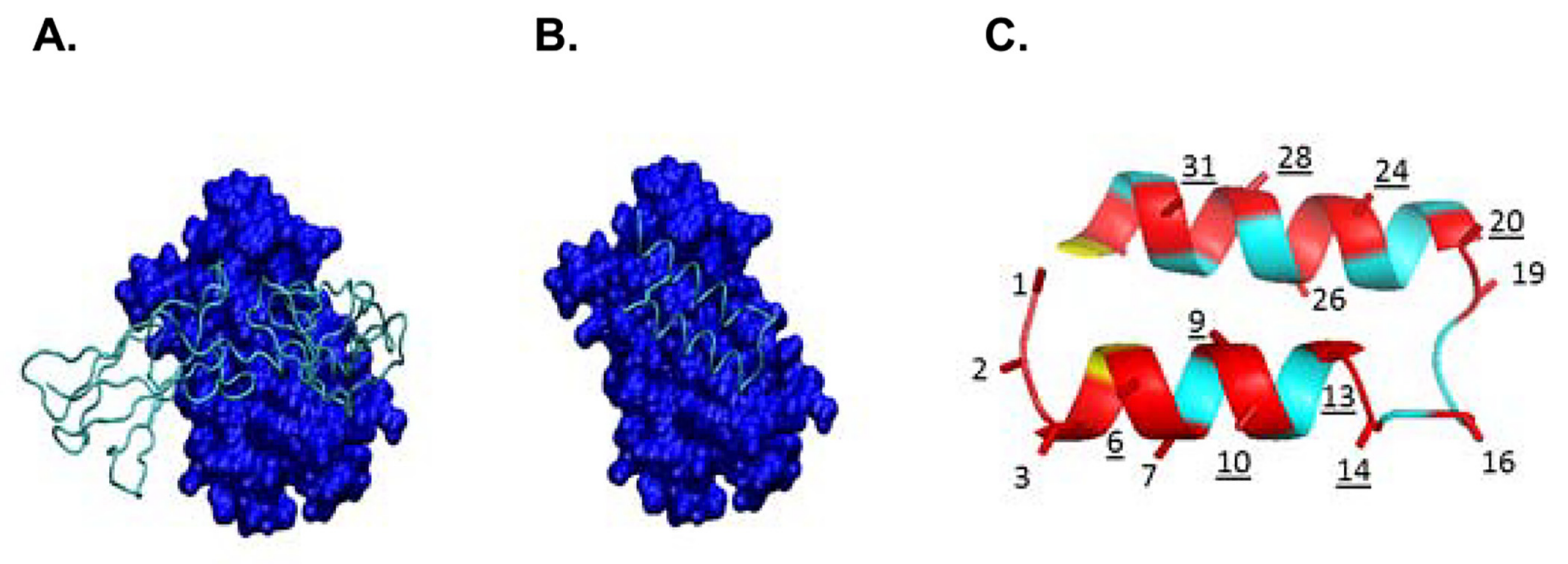
Structural models of IL-6, IL-6R and ZDC scaffold. **A.** Crystal structure of IL-6R (cyan tube) bound to IL-6 (blue surface model). Cα-Cβ vectors of interacting residues of IL-6R with IL-6 were used as a query. **B**. A model of ZDC (cyan tube) that matches Cα-Cβ vectors of IL-6R bound to IL-6 (blue surface model). **C.** Guided by the helical nature of the ZDC scaffold, residues (red) that were mutated in phage display and medicinal chemistry to optimize potency are shown. Underlined residue numbers are those that were a component of the initial random library. The remaining residues were explored during optimization with the goal of expanding the initial binding footprint of ZDC, including extending the termini (residues not shown).

From the crystal structure of the hexameric IL-6/IL-6R/gp130 complex [16] the Cα-Cβ vectors of IL-6R residues that contact IL-6 were used to form a query. The Cα-atom is anchored to the backbone and the Cβ-atom is anchored to the first side-chain atom [17,18]. The Cα-Cβ vector of IL-6R residues that are in contact with IL-6 are a measure of the topology of the interaction surface. These vectors were used as a query to find disulfide-rich peptides (DRPs) that share or match components of this same Cα-Cβ vector topology from a database of three dimensional structures of DRPs compiled from the protein databank [19] using the program Vectrix [20]. The resulting virtual hits were superimposed onto the original IL-6R Cα-Cβ query to derive a model of interaction with IL-6. Final scaffold selection was driven by the quality of the match, the degree of steric clash, if any, with IL-6, and the potential size of the interacting footprint with IL-6 (Fig 1B). The helix-loop-helix scaffold (Fig 1C), named ZDC in the protein-databank and Z34C in the original publication [21], was selected as a starting scaffold for lead optimization. This peptide is a 34-residue form of the 59-residue B-domain of the protein-A scaffold [21]. We hypothesized that disulfide-rich peptides that share surface Cα-Cβ topologies with the IL-6 binding surface on IL-6R have a higher probability of yielding hits that bind to IL-6.

### Phage display

Phage display [21,22] was used to select IL-6 binding phage particles from a library comprising up to 20^10^ theoretical sequences. Briefly, a DNA library encoding the ZDC scaffold, with positions on the scaffold (Q6, F9, Y10, L13, H14, E20, N24, A25, K28, R31) randomized with the NNK (N = A, T, C or G; K = G or T) codon (Fig 1C), was cloned into an M13 phagemid vector in frame with the M13 gene III. This process resulted in a library of phage expressing diversified ZDC sequences as pIII fusions. For library selection, 1 mL of ZDC phage particles in phosphate-buffered saline (PBS) containing 0.5% bovine serum albumin (BSA) were incubated with varying concentrations of biotinylated IL-6 target in solution. For the first round of panning, this step was carried out overnight at 4°C with rotation, while subsequent rounds were carried out for 2 h at room temperature. Target-bound phage were then captured using SA (streptavidin)-coated paramagnetic beads (Dynabeads® MyOne™ Streptavidin T1, Life Technologies, Grand Island, NY), after which the beads were extensively washed with PBS containing 0.1% Tween-20 to remove non-binding phage. The remaining phage particles were eluted from the beads through incubation with 10 mg/mL trypsin for 30 min, and amplified by infecting log phase XL1-Blue cells. The cultures were superinfected with VCSM13 helper phage and grown overnight at 30°C. The amplified phage particles were purified from culture supernatant by polyethylene glycol (PEG; average MW of 8000 Da) precipitation, and panning was repeated as above for a total of four rounds. Individual clonal analysis was undertaken on the output of the last round of panning through a combination of DNA sequencing and single-point IL-6 binding phage ELISA. IL-6 binding phage clones of interest were evaluated in a dose-dependent, competition format with soluble IL-6R to confirm inhibition. Based on the clonal analysis data, 16 initial sequences were selected for chemical synthesis and assay.

### Peptide synthesis, PEGylation, and purification

Peptides were synthesized using the Merrifield solid phase synthesis techniques on a 12 channel multiplex Symphony® peptide synthesizer (Protein Technologies, Inc., Tucson, AZ) and were assembled using O-Benzotriazole-N,N,N’,N’-tetramethyluroniumhexafluorophosphate (HBTU) and N,N-diisopropylethylamine (DIPEA) coupling conditions. Rink Amide MBHA resin (0.49mmol/g, 100–200 mesh, Catalog # RRZ005, Aapptec, Louisville, KY) was used for peptides with C-terminal amides and pre-loaded Wang Resin with N-α-Fmoc protected amino acids was used for peptides with C-terminal acids. The coupling reagents (HBTU and DIPEA premixed) and amino acid solutions were prepared in dimethylformamide (DMF) at a concentration of 100 mM. The peptides were assembled using standard Symphony® protocols. Pre-loaded Wang resin (250 mg,0.14 mmol, 0.56 mmol/g loading, 100–200 mesh) or MBHA resin (250 mg, 0.15 mmol, 0.6 mmol/g loading, 100–200 mesh) was placed in each reaction vial and washed twice with 4 mL of DMF followed by 2 x 10 min treatments with 2.5 mL of 20% 4-methylpiperidine/DMF (conditions for Fmoc deprotection). Either the Wang resin or the Rink Amide MBHA resin was then washed three times with DMF (4 mL), followed by addition of 2.5 mL of amino acid and 2.5 mL of a HBTU-DIPEA mixture. After 45 min of reaction with frequent agitation, the resin was filtered and washed three times with DMF (4 mL). This process was then repeated.

The coupling reaction was carried out twice for the first 25 amino acids and three times for the remaining amino acids. The assembled peptide on resin was then cleaved using a 2 h treatment with cocktail reagent K [23]. The cleaved peptides were precipitated in cold (0°C) diethyl ether, followed by washing two times with diethyl ether and air drying. The crude peptides were then submitted to an oxidation reaction in order to form the disulfide bridge. The crude peptide was dissolved in 50% acetonitrile/water at a concentration of 0.5 mg/mL. A saturated solution of iodine in methanol was added drop-wise until a yellow color persisted. Excess iodine was quenched by the addition of solid ascorbic acid until the solution became colorless. The resulting solution was purified by preparative reverse-phase HPLC: Phenomenex® Luna C18 column (10 μm, 300 Å, 250 x 21.2 mm) using buffer A (0.1% trifluoracetic acid (TFA) in water), buffer B (0.1% TFA in acetonitrile) gradient 33%-55% buffer B over 45 min, flow rate 20 mL/min, detection at 220 nm. Fractions containing the desired product were pooled and lyophilized with TFA to give a white solid.

Oxidized peptide (50 mg, 0.01 mmol) was dissolved in 3 mL of a 7:3 dimethyl sulfoxide (DMSO)-acetonitrile mixture. Activated 40 kDa branched PEG (PEG40Br, Catalog # GL2-400GS2, 360 mg, 0.009 mmol, Sunbright®, White Plains, NY), was added and followed by addition of 20 μL of DIPEA. The reaction mixture was stirred at room temperature for 8 h. Another aliquot of PEG40Br (240 mg, 0.006 mmol) and 16 μL of DIPEA were then added sequentially to the reaction mixture to help drive the reaction to completion (since the NHS- activated PEG can get hydrolyzed over time during the reaction and become un-reactive), and the reaction stirred for an additional 24 h at room temperature. The total PEG to peptide ratio was 1:1.5. The reaction was monitored by analytical HPLC. The reaction is site-specific, only occurring at the free amine of Lysine at position 29- the presence of peptide was monitored and no loss of peptide was observed after PEGylation. After the starting material had been consumed, the solution was poured into 20 mL of cold (0°C) diethyl ether, centrifuged and the diethyl ether decanted. The precipitate obtained was washed twice with cold diethyl ether. Excess PEG was removed by using strong anion exchange chromatography followed by purification using reverse phase HPLC. Anion exchange chromatography was performed using GE Source® 30Q resin on a 25 g column cartridge (Buffer A = 0.02 M bis-Tris pH ~6.5 in water and buffer B = 0.02 M bis-Tris pH ~6.5 + 1.0 M NaCl in water) in order to remove free PEG. The crude material (250 mg) was dissolved in 100% Buffer A and loaded onto the column. A gradient was performed with a flow rate of 15 mL/min as follows: (t, %B)—(0, 0); (10, 0); (25, 100); (30, 100). Fractions containing the desired product were pooled and lyophilized. The lyophilized solid was then dissolved in 0.2% acetic acid (AcOH) in water and purified by reverse phase HPLC on a Phenomenex® Jupiter C18 column (250 x 21.5 mm, 5μm, 300Å). The buffers used were: A = 0.2% AcOH in water, B = 0.2% AcOH in acetonitrile. Gradient: 20%-55% B over 30 min; flow rate: 20 mL/min. Fractions containing the desired product were pooled and lyophilized with 0.2% AcOH buffer to give the desired peptide. The PEGylated peptide did not lose its activity. Other activated PEGs were coupled to peptides. These PEG moieties included a 20 kDa linear PEG (PEG20L, Catalog # ME-200CS, Sunbright®), a 20 kDa branched PEG (PEG20Br; Catalog #GL2-200GS2, Sunbright®), and a 30 kDa linear PEG (PEG30L; Catalog # ME-300CS, Sunbright®). The same reaction procedure and purification methods were applied to these PEGylation reactions to obtain corresponding final PEGylated peptides with similar yields.

### Competition ELISA

Peptides were initially evaluated using a competitive enzyme-linked immunosorbent assay (ELISA). High binding 96-well microtiter plates (half-area, Corning Life Sciences, Corning, NY)) were coated with 0.1 μg per well of IL-6R (PeproTech, Rocky Hill, NJ) overnight at 4°C. The wells were then blocked for 2 h with a 1% BSA (Sigma, St. Louis, MO) solution containing 0.1% Tween-20 (Amresco, Solon, OH) and subsequently washed with wash buffer (PBS containing 0.1% Tween-20). Peptides were serially diluted in assay buffer (0.1% BSA, 0.05% Tween-20 in PBS) and incubated with a 50 pM biotinylated IL-6 (PeproTech) for 30 min at room temperature. This mixture was then transferred to an assay plate, and the plate incubated for 1 h. The plate was washed with wash buffer, and presence of bound IL-6 detected through addition of streptavidin poly-horseradish peroxidase conjugate (Pierce, Rockford IL). Plates were developed using a TMB substrate kit (Pierce) and stopped with 2N H_2_SO_4_. Inhibition of complex formation was measured as a reduction in absorbance at 450 nm.

### pSTAT3 HTRF assay

Peptides were serially diluted in medium containing 10% fetal bovine serum (FBS)/4% DMSO, then added to recombinant IL-6 (Xtal Biostructures, Inc., Natick, MA) at 28.2 ng/mL and the resulting plate was incubated at room temperature with shaking for 2.5 h at 37°C. U937 human histiocytic lymphoma cells (CRL1593, ATCC, Manassas, VA), were plated in media in a volume of 80 μL in a 96-well V-bottom tissue culture plate at 1.25x10^6^ cells/mL and 16 μL of the peptide/IL-6 solution was added to the cells. After incubation for 12 min at 37°C the cells were centrifuged at 4°C (1400 rpm for 5 min) and the media was removed from the plates. Cells were placed on ice and 50 μL lysis buffer (Cisbio Assays, Bedford MA) was added. Phosphorylation of STAT3 was detected as described by the provider (Cisbio Assays). Briefly, cells were lysed for 30 min at room temperature and then 16 μL of lysates was added to a 384-well Proxiplate (Perkin Elmer, Waltham, MA). Anti-STAT3-d2+anti pSTAT-3-K detection cocktail (4μL; Cisbio Assays) was then added to the wells and the plate covered with a plate sealer and returned to 4°C overnight. Plates were then read with an Envision plate reader at ex 340, em 615 and 665 nm. Species selectivity assays against mouse, rat and/or cynomolgus IL-6 (all purchased from R&D Systems, Minneapolis, MN) were conducted similarly to the pSTAT3 experiments except Balb/c-3T3 (ATCC) cells were used. PN-2921-mediated inhibition of an IL-6 concentration response in U937 cells was carried out by incubating various concentrations of human IL-6 (comprising a concentration response curve) with constant amounts of peptide for 2.5 h at 37°C. These mixtures were then added to U937 cells for 15 min at 37°C and pSTAT3 was detected as described above. Data were analyzed and graphed using GraphPad Prism software.

### B9 cell proliferation assay

Mouse hybridoma B9 cells (ATCC) were maintained in RPMI 1640 culture medium supplemented with 100 pg/mL human recombinant IL-6 (Xtal Biostructures, Inc.). B9 cells were cultured without IL-6 for 3 days at a concentration of 200,000 cells/mL in medium containing 5% FBS and penicillin/streptomycin (50 Unit/mL and 50 μg/mL, resp. Catalog # 15070–063, Gibco). On the day of the experiment, cells were prepared by washing once in RPMI 1640 containing 5% FBS, followed by centrifugation at 300 x g for 5 min. Cell number and viability were determined by trypan blue exclusion. Cells in 50 μL media (5000 total cells) were dispensed into a 96-well tissue culture-treated plate containing 25 μL of inhibitors prepared at varying concentrations and 25 μL of IL-6. Final DMSO concentration was 0.1–0.2%. The plate was incubated for 72 h at 37°C in a humidified 5% CO_2_ atmosphere, then 0.2 μCi/well of 1mCi/ml ^3^H-thymidine (Catalog # ART 1241, American Radiolabeled Chemicals, St. Louis, MO) was added for the final 18 h. After 72 h, cells were transferred to a 96-well filter plate (Catalog # MADVNOB, Millipore, Billerica, MA) that was pre-washed with PBS. Filters were washed with 150 μL of water 6 times, and dried at 50°C for 30 min; 80 μL of scintillation fluid was added (UltraGold MV, Catalog # 6013159. PerkinElmer) and radioactivity was quantitated using a Microbeta Plate Reader (Perkin Elmer). Data were analyzed and graphed using GraphPad Prism software (GraphPad Software).

### Whole human blood assay

Freshly collected heparinized human whole blood was obtained from human donors supplied by the CRO (Research Blood Components, Brighton, MA). Blood was used from six human donors to provide sufficient volume of starting material and to allow representation of a more heterogenous cell source. Written informed consent was obtained from the donors in accordance with the New England IRB approved protocol NEIRB #04–144. PEGylated peptide, or vehicle control, were incubated with IL-6 for 2.5 h at 37°C and then added to heparinized human whole blood (pooled from 6 donors) for 15 min at 37°C. The final concentration of IL-6 was 2.5 ng/mL. Red blood cells (RBCs) were lysed with 500 μL RBC Lysing Buffer (Sigma), and samples mixed gently for 10 min at 37°C. Samples were centrifuged at 400 x g for 5 min to pellet cells and the supernatants were removed. Samples were washed with wash buffer (4% BSA/PBS, Gibco, Thermo-Fischer Scientific, Grand Island, NY), then centrifuged at 400 x g for 5 min to pellet cells. Cell pellets were permeabilized for 10 min at 37°C in 2% formaldehyde in PBS (Polysciences Inc., Warrington, PA) and then fixed in ice cold methanol for 30 min. Cells were washed once with wash buffer and then stained with anti-phosphorylated STAT3 (Y705) antibody conjugated to Alexa Fluor 488 (BD Pharmingen, Billerica, MA) diluted in wash buffer, final volume 100 μL. Samples were incubated on ice for 30 min then washed twice and the final cell pellets re-suspended in 400 μL IF Buffer (Hank's Buffered Saline Solution, 2% Fetal Calf Serum, 10 mM Hepes, 0.2% Sodium Azide, Gibco). A Guava instrument (Millipore) using Easycyte software was used to collect and analyze data.

### Comparative PK studies of PEGylated peptides in rats

Male rats for pharmacokinetic studies were obtained from Harlan Laboratories (South Easton, MA. Anti-IL-6 peptides were administered to rats as a single subcutaneous dose at 0.23 mmol/kg (a dose determined from previous pilot studies) and blood samples were collected at fixed time intervals for up to 1 week post-dose. For all time points, blood samples were collected via retro-orbital eye bleed or exsanguination via abdominal aorta into tubes containing K_2_EDTA and were processed into plasma, which was placed in Eppendorf Protein LoBind tubes and stored frozen at approximately -70°C until quantitative bioanalysis.

The dosing solutions were formulated just prior to administration, in sterile 1X PBS with 0.1% BSA. Plasma sample extraction was performed by aliquoting 100 μL of each rat plasma sample into a standard 96-well plate. To each well, 400 μL of 5% formic acid in acetonitrile (containing 200 ng/mL of internal standard) was added. The plate was vortexed at high speed for approximately 10 min and then centrifuged for 15 min, 4700 x g at 4°C. Supernatant (425 μL) was transferred into a clean 96-well plate and evaporated to dryness under nitrogen at approximately 50°C. The extracts were then reconstituted in 50 μL of 1% formic acid in 60% acetonitrile.

Reconstituted extracts (5 μL) were analyzed by liquid chromatography-tandem mass spectrometry (LC-MS/MS) with positive electrospray ionization (QTrap 5500, AB SCIEX, Framingham, MA). Chromatographic separation (Acquity UPLC, Waters Corp., Milford MA) was achieved using a gradient under reverse phase conditions using either a 1.9 μm Thermo Hypersil Gold (2.1 x 50 mm) or a 1.7 μm Waters Acquity BEH300 (2.1 x 50 mm) column. Mobile phases consisted of 0.1% formic acid in water and 0.1% formic acid in acetonitrile:water (90:10, v:v) set at a flow rate of 0.8 mL/min over a total run time of five minutes.

Data were collected and processed using Analyst Software version 1.5.2 (AB SCIEX). Matrix-matched standard curves were generated using peak area ratios of analyte to internal standard vs. concentration. A 1/x weighted linear regression was performed to generate the relationship between response and concentration. Non-compartmental PK analysis was performed with the plasma concentration data to determine the PK parameters using Phoenix WinNonlin v6.3 (Certara, Inc.).

### Comparative PK studies of PN-2921 in mice, rats and monkeys

The PEGylated peptide, PN-2921, was administered as a single intravenous (IV) or subcutaneous (SC) dose at 0.1 umol/kg to male cynomolgus monkeys. Blood samples were collected from 3 monkeys per time point at fixed intervals for up to two weeks post-dose. PN-2921 was also administered as a single IV or SC dose at 0.23 μmol/kg to male ICR mice and male Sprague Dawley rats. Blood samples were collected from 3 mice per time point or from 4 rats per time point at fixed intervals for up to one week post-dose.

The dosing solutions were formulated on the day of dosing, just prior to administration, in sterile 1X PBS with 0.1% BSA. Both intravenous (IV) and subcutaneous (SC) dosing solutions were formulated as a single preparation. For all time points, blood samples were collected into tubes containing K_2_EDTA and processed into plasma which was placed in Eppendorf Protein LoBind tubes and stored frozen at approximately -70°C until quantitative bioanalysis. Plasma samples were extracted using protein precipitation followed by overnight trypsin digestion to generate a 15 amino acid peptide surrogate of PN-2921 followed by analysis by LC-MS/MS. To accomplish this, PN-2921 was extracted from 100 μL of plasma by protein precipitation using 400 μL acetonitrile with 5% formic acid in the presence of an internal standard. After being vortexed and centrifuged for 10 minutes at 16,000 x g, 425 μL of the supernatant was then transferred to a clean plate and evaporated under nitrogen with a plate temperature of 55°C. The extracts were reconstituted in 300 μL of digestion buffer (10% methanol, 90% 50 mM ammonium bicarbonate) and then 50 μL of 40 μg/mL trypsin in 50 mM ammonium bicarbonate was added for overnight digestion at 37°C on a plate shaker. The reaction was stopped with 40 μL of 10% formic acid and 50% methanol and evaporated under nitrogen until dry. The extracts were then reconstituted in 50 μL of 1% formic acid in 60% acetonitrile.

Reconstituted samples (10 μL) were analyzed by LC-MS/MS with positive electrospray ionization (Qtrap 5500, AB SCIEX). Chromatographic separation (Acquity UPLC, Waters Corp) was achieved using a gradient under reverse phase conditions using a 1.9 μm Thermo Hypersil Gold (2.1 x 50 mm) column. Mobile phases consist of 0.1% formic acid in water and 0.1% formic acid in acetonitrile:water (90:10, v:v) set at a flow rate of 1.0 mL/min over a total run time of six minutes. Data were collected and processed using Analyst Software version 1.5.2 (AB SCIEX) as described above.

### IL-6-induced biomarker studies in mice and monkeys

Male Balb/c mice (Harlan Laboratories), 7–8 week old, weighing 23–27 g were housed 4 per cage for at least 1.5–3 weeks prior to testing. Mice received an SC administration of 0.0023, 0.023, 0.23 or 2.3 μmol/kg of peptide or 0.04 μmol/kg control anti-recombinant human (rh) IL-6 antibody (Catalog # MAB206, R & D Systems) 24 h prior to an intra-peritoneal administration of 125 ng (5 μg/kg) of human IL-6. Anti-rhIL-6 antibody (control) and PEGylated peptides were suspended directly in PBS/0.1% BSA. For preparation of PEGylated peptides solutions, the method included 10–60 s of sonication in a sonication water bath to ensure proper solubilization of the peptides.

Recombinant human IL-6 (Catalog # 28–212, Xtal Biostructures, Inc.,) was prepared in PBS/1% BSA and administered by intra-peritoneal injection in a volume of 100 μL at a dose of 125 ng (ca. 5 μg/kg). Mice were euthanized 4 h post IL-6 administration using a CO_2_ overdose and blood was obtained via the abdominal aorta. Blood was collected into K_2_EDTA tubes for preparation of plasma and subsequent evaluation of SAA levels by ELISA (Catalog # KAA0021, Life Technologies/Tridelta).

Studies in monkeys were performed by Xenometrics LLC (Stilwell, KS, USA). Adult, male cynomolgus monkeys (*Macaca fascicularis*) (6 per group) were randomly assigned to dose groups based on body weight (manually allocated to achieve similar group mean weight) and using the supplier-applied permanent tattoo identification. Each monkey received a single subcutaneous injection of either vehicle (PBS) or PN-2921 in vehicle administered at 2.3 μmol/kg (100 mg/kg) in a volume of 2 mL/kg. Twenty four hours after pre-treatment with vehicle or PN-2921, animals received a single subcutaneous injection of recombinant human IL-6 (rhIL-6; R&D Biosystems) in PBS containing 1% w/v BSA with low endotoxin (Sigma) at 0.6 μg/kg in a volume of 1 mL/kg. Immediately before administration of vehicle or PN-2921 and at 24 h after administration of rhIL-6, blood samples were collected via cephalic or saphenous vein into tubes containing K_2_ EDTA. Plasma was prepared by spinning tubes containing whole blood at 2700 x g for 10 min at 4°C. Plasma samples were prepared in aliquots and stored frozen at –80°C until analyzed for levels of C reactive protein (CRP), serum amyloid A (SAA), and PN-2921. CRP levels were evaluated using a Randox Ximola clinical analyzer. SAA levels were measured by ELISA (Tridelta, Multispecies Catalog # TP-802). Statistical analysis was performed using Tukey’s multiple comparisons test. Data were analyzed and graphed using GraphPad Prism (Graphpad Software, Inc. San Diego, CA). The animals were returned to the colony at Xenometrics after the completion of the study.

### Animal housing and care

Studies with mice and rats were conducted by Ironwood Pharmaceuticals (Cambridge, MA). Ironwood is fully accredited by the Association for Assessment and Accreditation of Laboratory Animal Care International (AAALAC). All procedures involving rodents were conducted humanely and performed by trained and experienced personnel. All studies involving rodents were reviewed and approved by the Ironwood Institutional Animal Care and Use Committee (IACUC). Rats and mice (Harlan Labs) were housed in a temperature-controlled environment with ad libitum access to rodent chow (Harlan Teklad) and water. The animals were group housed and provided with enrichment. Blood was drawn from rats and mice under isoflurane anesthesia. Rats and mice were euthanized by CO_2_ inhalation in accordance with AVMA Guidelines.

Studies with cynomolgus monkeys were conducted at Xenometrics, a CRO fully accredited by AAALAC and registered with the United States Department of Agriculture (USDA). Cynomolgus monkeys were individually housed in cages (7.8 ft^2^), provided with ad libitum access to food and water, and supplemented with fruit and snacks at least once daily. Animals were exercised and participated in enrichment programs during the study. Enrichment included but was not limited to: cage perches, swings, mirrors, toys to manipulate, scent stimulation, watching videos and social interaction with technicians. All procedures involving non-human primates were conducted humanely and performed by trained and experienced personnel. Animals were habituated and trained for injection and blood draw procedures. All studies followed protocols reviewed and approved by the Xenometrics IACUC and these studies were not initiated until they were reviewed and approved by the IACUC.

## Results and Discussion

### Hit identification and lead optimization

To identify a starting point for an IL-6 binding peptide, we took advantage of the crystal structure of the hexameric IL-6/IL-6R/gp130 complex [16] and a large virtual library of DRPs from the protein databank [19]. Cα-Cβ vectors of key IL-6 interacting residues in IL-6R (Fig 1A) were generated where the Cα-atom was anchored to the backbone and the Cβ-atom was anchored to the first side-chain atom. These vectors were used as a query in Protagonist’s Vectrix program [20] to find peptides that share or match this same Cα-Cβ vector topology. These computational searches yielded several potential scaffolds that matched surface features of IL-6R and were predicted to be suitable scaffolds to interact with IL-6. The most promising scaffold selected for further investigation was a 34 amino acid peptide (Fig 1B and 1C; complete sequence shown in S1 Table) referred to as ZDC in the protein-databank, and Z34C in the original publication [21]. Structurally, ZDC is a disulfide-stabilized peptide containing two antiparallel α-helices (Fig 1C) [21].

The ZDC peptide had no inhibitory activity against IL-6 itself (Table 1) but was used as a scaffold for a phage display approach [22,24]. Modeling predicted ten residues of ZDC that would likely contact with the IL-6 surface (Fig 1C), and these residues (positions 6, 9, 10, 13, 14, 20, 24, 25, 28 and 31) were fully randomized to generate a phage display library. After four rounds of panning against immobilized IL-6, individual phage clones were functionally characterized and sequenced. Based on the data generated, 16 initial sequences were selected for chemical synthesis and evaluated in an IL-6 –IL-6R competition ELISA. PN-1454 (Table 1 and S1 Table for complete sequence) was among the most active peptides with an IC_50_ of 12 μM. These early peptide hits were able to disrupt IL-6/IL-6R interactions in the ELISA assay, but lacked the potency to antagonize IL-6-induced pSTAT3 in intact U937 cells (Table 1), a human monocytic cell line known to express IL-6R [25].

**Table 1:** Peptide Sequences and Anti-IL-6 Activity of Key Peptides in the Development of PN-2921.

Peptide	Scaffold position and amino acid																								Activity	
	-2	-1	0	1	2	3	4	6	7	9	10	13	14	16	19	20	24	25	26	28	29	31	33	35	ELISA IC_50_ (nM)	Cell IC_50_ (nM)
**ZDC**				F	N	M	Q	Q	R	F	Y	L	H	P	N	E	N	A	K	K	S	R	D		>20000	>20000
**PN-1454**				F	N	M	Q	I	R	L	M	F	L	P	N	S	W	G	K	D	S	N	D		12000	>20000
**PN-1484**				F	N	L	Q	I	R	L	L	F	L	P	N	S	W	G	K	D	S	N	D		645	>20000
**PN-1974**				F	D	hL	D	I	H	L	L	F	L	P	T	E	W	E	K	D	K	N	E	E	126	3284
**PN-2519**			Q	S	D	Cha	D	I	H	L	L	F(4-F)	L	P	T	E	W	E	R	Gla	K	N	E	E	ND	3.7
**PN-2729**	S	W	Q	S	D	Cha	D	I	H	L	L	F	L	K-Ac	T	E	W	E	R	D	K	N	E	E	ND	1.0
**PN-2921**	S	W	Q	S	D	Cha	D	I	H	L	L	F	L	K-Ac	T	E	W	E	R	D	K PEG 40Br	N	E	E	ND	0.4

Subset of residues (position and specific amino acids) mutated on the original ZDC scaffold and key compounds in the development of PN-2729 and its PEGylated analog, PN-2921. All peptides are N-terminally acetylated and all peptides were C-terminal α-amidated except for PN-2519, PN-2729, and PN-2921. Amino acid positions that were randomized by phage display are indicated in bold. IC_50_ values (n = 2 or more) of peptides in an IL-6 ELISA and an IL-6-induced pSTAT3 assay in U937 cells are shown.

ND = not determined.

hL = homoleucine

Cha = cyclohexyl-L-alanine

F(4-F) = 4-fluoro-L-phenylalanine

Kac = Nε-acetyl-L-Lys

Gla = gamma-carboxyglutamic acid.

KPEG40Br = 40 kDa branched PEG.

Hit and lead optimization was carried out using a combination of molecular modeling, phage display and medicinal chemistry. Early in the optimization process it was noted that PN-1454 contained two Met residues which could be readily oxidized resulting in potential chemical stability issues. PN-1484 was designed with M3L and M10L substitutions (Table 1), which resulted in ~20-fold improvement in IC_50_ in the competitive ELISA. Guided by the helical nature of the ZDC scaffold (Fig 1C), additional phage display libraries and medicinal chemistry approaches were employed, with a focus on mutating select positions (2, 3, 4, 7, 19, 29, 33 and 35) predicted to interact with IL-6 to expand the footprint of interaction of PN-1454. These approaches resulted in PN-1974 (Table 1), which had an IC_50_ of 126 nM in the competitive ELISA and an IC_50_ of 3.3 μM in a cell-based assay based on inhibition of IL-6-induced STAT3 phosphorylation in U937 cells. PN-1974 was among the earlier peptides which exhibited measurable potency in the cell-based assay. Molecular modeling indicated the presence of a positively charged surface patch on IL-6 in close proximity to both helical termini of the scaffold (Fig 1B), providing another opportunity to increase the binding footprint. The N-terminus was therefore extended by the addition of Q0 and S1 to exploit this potentially favorable electrostatic interaction (Table 1). An exploration of hydrophobic interactions in the N-terminal helix of the ZDC scaffold resulted in the selection of cyclohexyl-L-alanine (Cha) at position 3. These three changes, in addition to replacing the C-terminal amide functionality to the acid (which was a feature present in all peptides up to PN-1974) led to the identification of PN-2519, a peptide with ~1000-fold improvement compared to PN-1974 in cell-based activity (Table 1). Several additional residue substitutions present in PN-2519 had no additional effect on potency (F13 to 4-fluoro-L-phenylalanine and K26 to R). Concurrently, a phage display strategy was employed to optimize the N-terminus; these results corroborated the Q0 and S1 findings and yielded an additional two residues, S-2 and W-1 in PN-2729. Throughout the SAR process, improvements in potency at any one position were often modest, but when combined with improvements at other positions led to significant improvements in overall potency. Complete sequences of all peptides shown in Table 1 can be found in S1 Table.

### Impact of PEGylation on potency and PK

**Fig 2:**
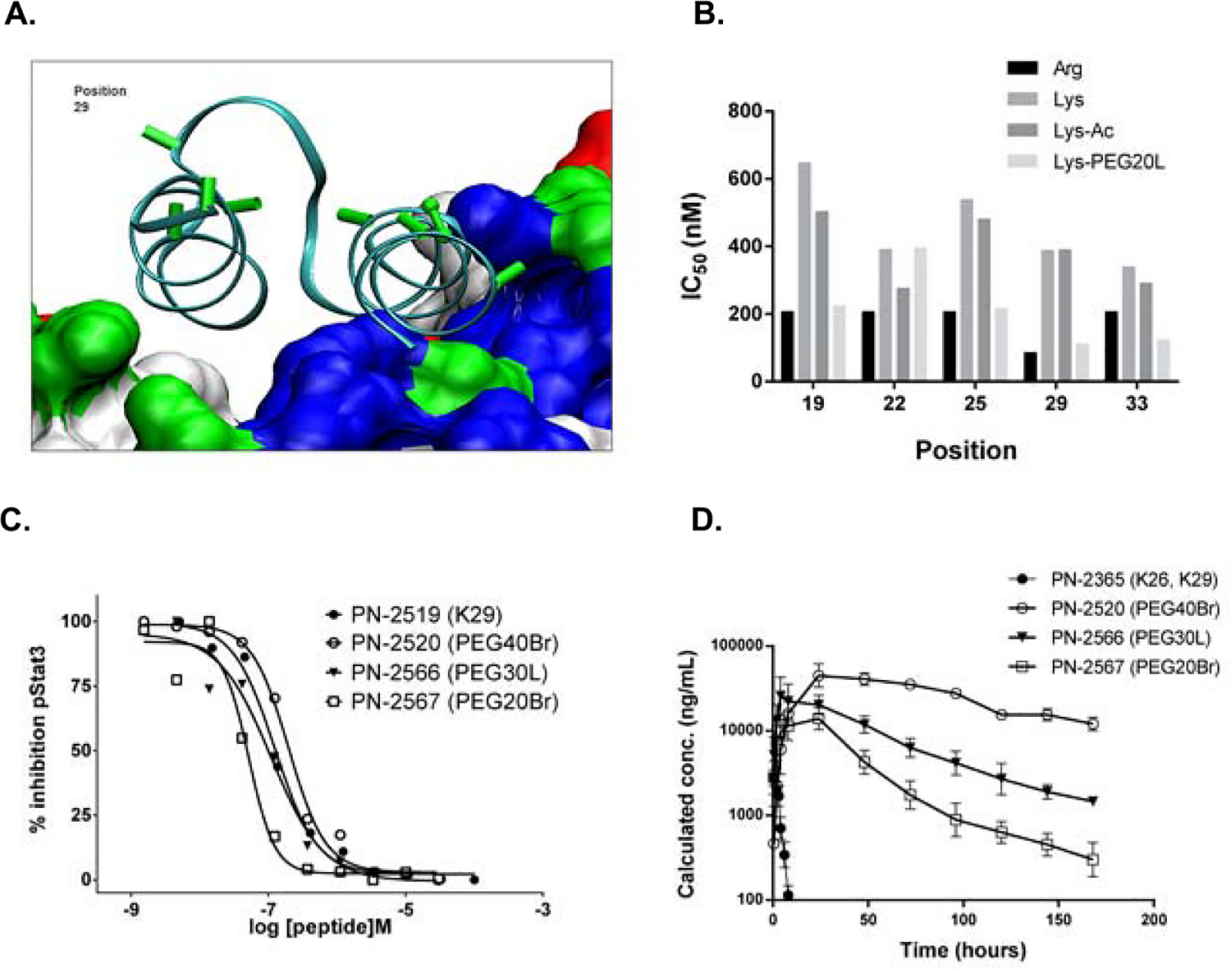
Determination of PEGylation site and effect of PEGylation on IC_50_ and plasma exposure. **A.** Ribbon diagram of ZDC (in cyan) bound to IL-6. Tolerated PEGylation sites on ZDC are indicated in green, and the final PEGylation site, position 29, is labeled. **B.** Five positions on the non-binding exoface of PN-2171 (sequence shown in S1 Table) were scanned with Lys, Nε-acetylated Lys (Lys-Ac), and LysPEG20L for their effect on IL-6 induced pSTAT3 in U937 cells. IC_50_ values are averages of n = 2 determinations. **C.** Effect of PEG molecular weight on the activity of peptides. PN-2519 is the non-PEGylated parent, PN-2520 contains a PEG40Br, PN-2566 contains PEG30L and PN-2567 contains a PEG20Br, all PEGylated at position 29. **D**. PK analysis in rats of peptide analogs with different molecular weight PEG moieties. In this experiment, the non-PEGylated comparator is PN-2365 (a PN-2519 analog with K26, K29). Peptides were administered by SC injection at 0.23 μmol/kg and plasma samples were taken at the indicated time points.

The remaining amino acids at positions 16, 26 and 29 of PN-2729 were optimized during evaluation of potential sites for PEGylation. It was anticipated, and indeed observed (Fig 2D), that an unmodified constrained peptide (PN-2365; S2 Table) would be cleared rapidly upon systemic administration. Since the intent was to develop an anti-IL-6 peptide that had prolonged PK, a PEGylation strategy was adopted. PEGylation of peptides and proteins has been extensively utilized to enhance PK properties [26]. To enable site-specific PEGylation, peptide analogs were required that had only one Lys (free amine) for acylation with activated PEG. Consequently Lys residues at position 26 and 29 were changed to Arg, and cell-based assays suggested that these changes were relatively well tolerated. R26 in PN-2519 was derived from this analysis (Table 1).

Since Lys to Arg did not have a significant impact on potency, a systematic incorporation of Lys was explored at the potential sites of the scaffold for PEGylation. Guided by existing SAR and molecular modeling (Fig 2A), a Lys, Nε-acetylated Lys (Lys-Ac), and LysPEG20L scan at several select positions was undertaken on the non-binding exoface of PN-2171, a close analog of PN-2519 (sequence shown in S1 Table). As illustrated in Fig 2B, substitution of Arg with Lys resulted in 1.5–3 fold decrease in IC_50_ values across the tested sites; acetylation of the Lys yielded similar potencies to the Lys residue. Interestingly, addition of a PEG20L moiety at all positions except position 22 improved potency and returned the activity to that seen with the Arg analog. Overall, PEGylation of residues predicted to be solvent exposed and not in the binding interface was well tolerated. Related studies found that acetylated Lys was unexpectedly more potent at position 16, resulting in K-Ac16 in PN-2729 (Table 1).

To confirm that potency could be maintained with increased steric bulk, PEG20Br, PEG30L, and PEG40Br analogs were prepared using position 29 as the site of PEGylation. The antagonist activity of these peptides, PN-2519 (K29), PN-2520 (PEG40Br), PN-2566 (PEG30L), and PN-2567 (PEG20Br) in the IL-6 pSTAT3 assay in U937 cells was comparable (Fig 2C and S2 Table for sequences). As expected, a pronounced impact of PEG size on PK properties was observed (Fig 2D and S3 Table). The non-PEGylated comparator peptide, PN-2365 (K26, K29), was rapidly cleared and had a PK half-life of 3.4 h, in the range of other non-PEGylated peptides that were characterized. Systemic exposure and specifically t_1/2_ increased with increasing PEG size, from 28.9 h with 20 kDa branched PEG (PN-2567) to as long as 63.3 h with 40 kDa branched PEG (PN-2520). The increased exposure was largely driven by decreased clearance, consistent with published studies showing that a PEG mass of approximately 40 to 50 kDa is required to slow the glomerular filtration rate of molecules [26]. The outcome from these studies was the selection of the PEG40Br and position 29 as the optimal moiety and site for PEGylation.

### In vitro characterization of PEGylated peptide

**Fig 3:**
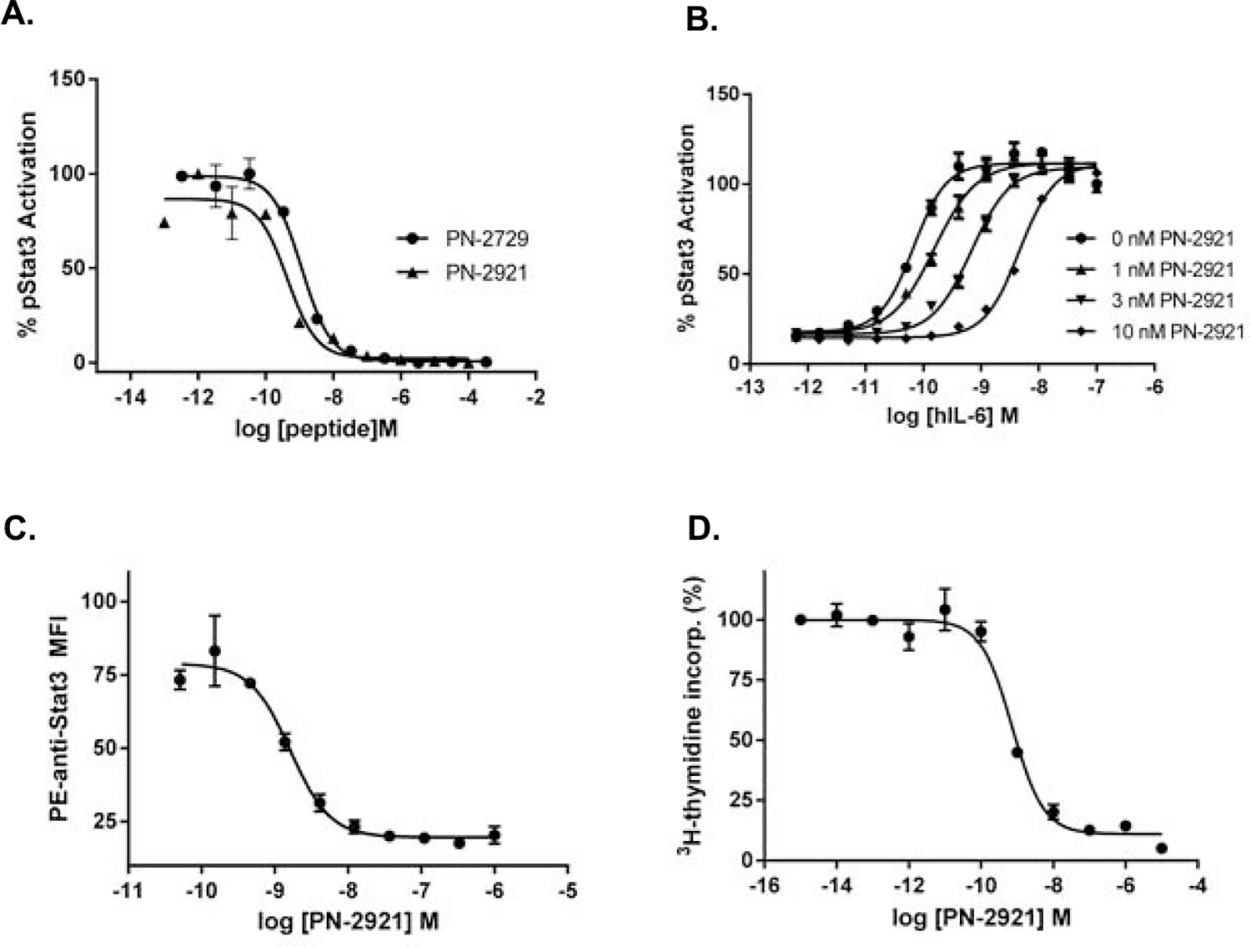
PN-2921 demonstrates potent anti-IL-6 activity in vitro. **A.** In vitro potency of PN-2729 and PN-2921 was determined by the ability to inhibit IL-6-induced pSTAT3 in U937 cells. Peptides were incubated with IL-6 for 2.5 h before cell treatment. The observed IC_50_ values in this representative experiment were 1.2 nM for PN-2729 and 0.4 nM for PN-2921. **B.** PN-2921 dose-dependently right-shifted the IL-6-induced pSTAT3 concentration response curve in U937 cells. **C**. PN-2921 decreased IL-6-induced pSTAT3 in monocytes. Peptides or vehicle control were incubated with IL-6 for 2.5 h at 37°C and then added to freshly collected heparinized human whole blood (pooled from six donors) for 15 min at 37°C. pSTAT3 was quantitated by flow cytometry. **D.** PN-2921 inhibited rhIL-6-induced proliferation in mouse B9 cells. B9 cells were incubated with rhIL-6 and varying concentrations of PN-2921 for 72 h at 37°C, with 0.2 μCi/well of ^3^H-thymidine present for the last 18 h.

The addition of a PEG40Br to position 29 of the most potent analog, PN-2729, resulted in PN-2921 (Table 1). PEGylation of PN-2729 at position 29 did not decrease potency, but in fact PN-2921 was consistently more potent at inhibiting IL-6-induced pSTAT3 in U937 cells than its non-PEGylated parent, PN-2729 (Fig 3A, Table 1). Further characterization of PN-2921 in the U937 cell assay demonstrated that increasing concentrations of PN-2921 potently shifted the IL-6-induced pSTAT3 concentration response curve to the right (Fig 3B). The inhibitory potency of PN-2921 was assessed in human whole blood monocytes in order to examine its activity in a more physiologically relevant cell; freshly isolated human monocytes express significant levels of IL-6R [27]. Similar to the observations in U937 cells, PN-2921 inhibited IL-6-induced pSTAT3 in human monocytes with low nM potency (Fig 3C). As expected of an IL-6 antagonist, PN-2921 inhibited biological functions downstream of IL-6 signaling: PN-2921 potently inhibited IL-6-induced proliferation of B9 mouse lymphoma cells with an average IC_50_ of 0.7 nM (Fig 3D). Average IC_50_ values for the inhibition of IL-6-induced cellular responses by PN-2921 are summarized in Table 2 and demonstrate that the potency of PN-2921 to inhibit IL-6 signaling is comparable across a range of cell types and assays. While not as potent as affinity matured anti-IL-6 mAbs such as siltuximab (with a potency in the pM range) [28], the low nM potency of PN-2921 is well within the range of many approved therapeutics.

**Table 2:** Summary of inhibitory Activity of PN-2921.

Assay	Cells	Average IC_50_ (nM)
Inhibition of IL-6-induced pSTAT3	U937	0.4
Inhibition of IL-6-induced pSTAT3	Human monocytes	3.1
Inhibition of IL-6-induced proliferation	B9	0.7

Average IC_50_ values for the activity of PN-2921 to inhibit IL-6-induced pSTAT3 signaling in U937 cells (n = 23), IL-6-induced inhibition of human whole blood monocytes (n = 6), and IL-6-induced proliferation in B9 cells (n = 3).

The ability of PN-2921 to inhibit pSTAT3 in response to IL-6 orthologs from different species was assessed in murine Balb/c-3T3 cells, which, unlike human IL-6R-expressing cells, respond to more distant IL-6 orthologs. PN-2921 was approximately three orders of magnitude less potent in inhibiting IL-6- induced pSTAT3 elicited by IL-6 orthologs from rodents as compared to that elicited by the human ortholog (Table 3). This is not surprising given that human IL-6 shares only 39%, and 42% identity with rat and mouse IL-6, respectively. In contrast, PN-2921 was equally effective at inhibiting cynomolgus monkey IL-6-induced signaling as human IL-6; this would be expected since cynomolgus and human IL-6 share 98% identity. Species cross-reactivity is an important consideration with mAbs and frequently complicates assessment of activity in preclinical animal studies [28].

**Table 3:** Inhibitory Activity of PN-2921 Against IL-6 From Other Species.

IL-6 Species	Average IC_50_ (nM)
Human	6.5
Cynomolgus monkey	6.7
Rat	5619
Mouse	>10000

In vitro potency of PN-2921 was determined by its ability to inhibit pSTAT3 signaling in Balb/c-3T3 cells induced by IL-6 from cynomolgus monkey, rat or mouse. IC_50_ values are averages of n = 2 experiments.

### PK and pharmacodynamic profile of PN-2921

**Fig 4:**
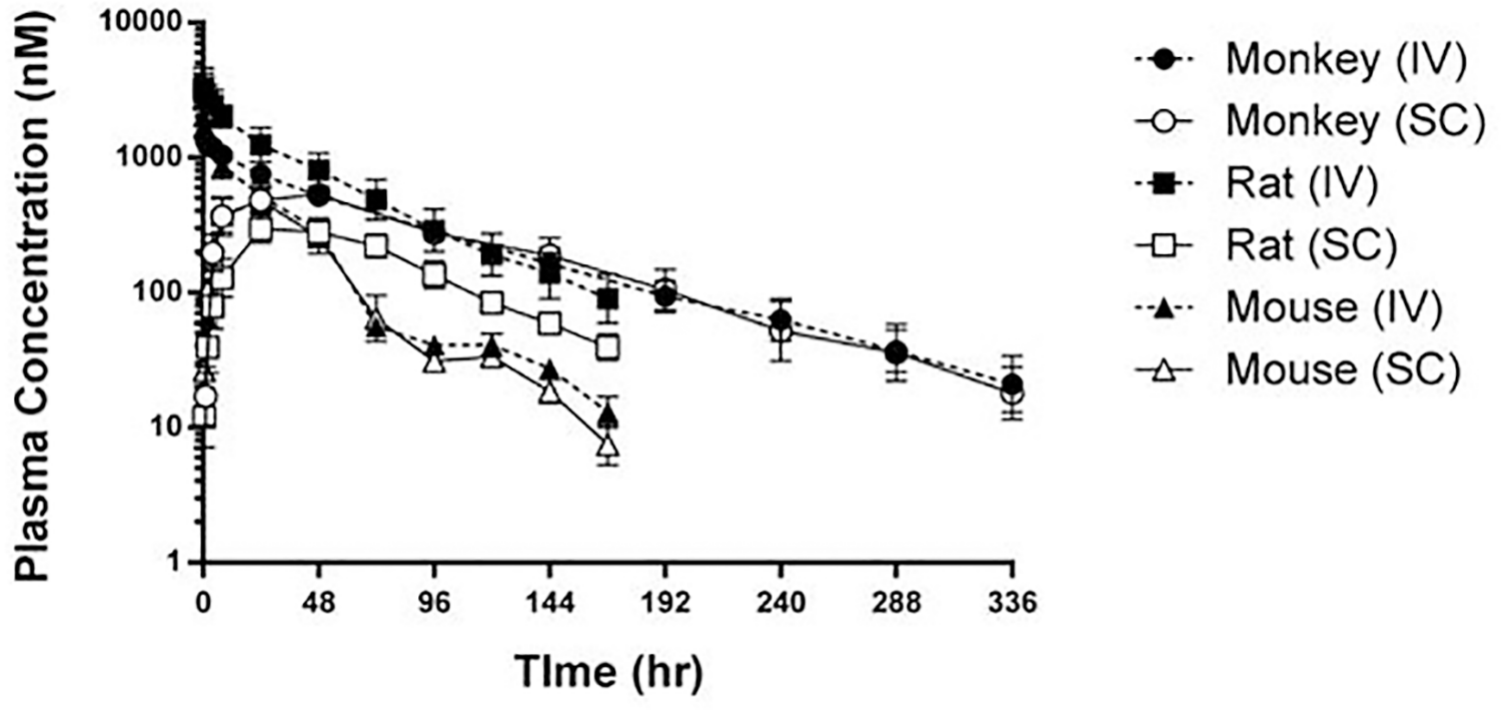
PN-2921 demonstrates long-lived exposure in multiple species. PN-2921 was dosed in male ICR mice, male SD rats and male cynomolgus monkeys by IV and SC routes. Both mouse and rats were dosed at 0.23 μmol/kg, while monkeys were dosed at 0.1 μmol/kg. Plasma samples were collected for 168 h (7 d) for both rats and mice, while monkey plasma samples were collected for 336 h (14 d). Samples were analyzed by LC-MS/MS. Mouse and rat data were normalized to 0.1 μmol/kg for the purpose of comparison to monkey.

**Fig 5:**
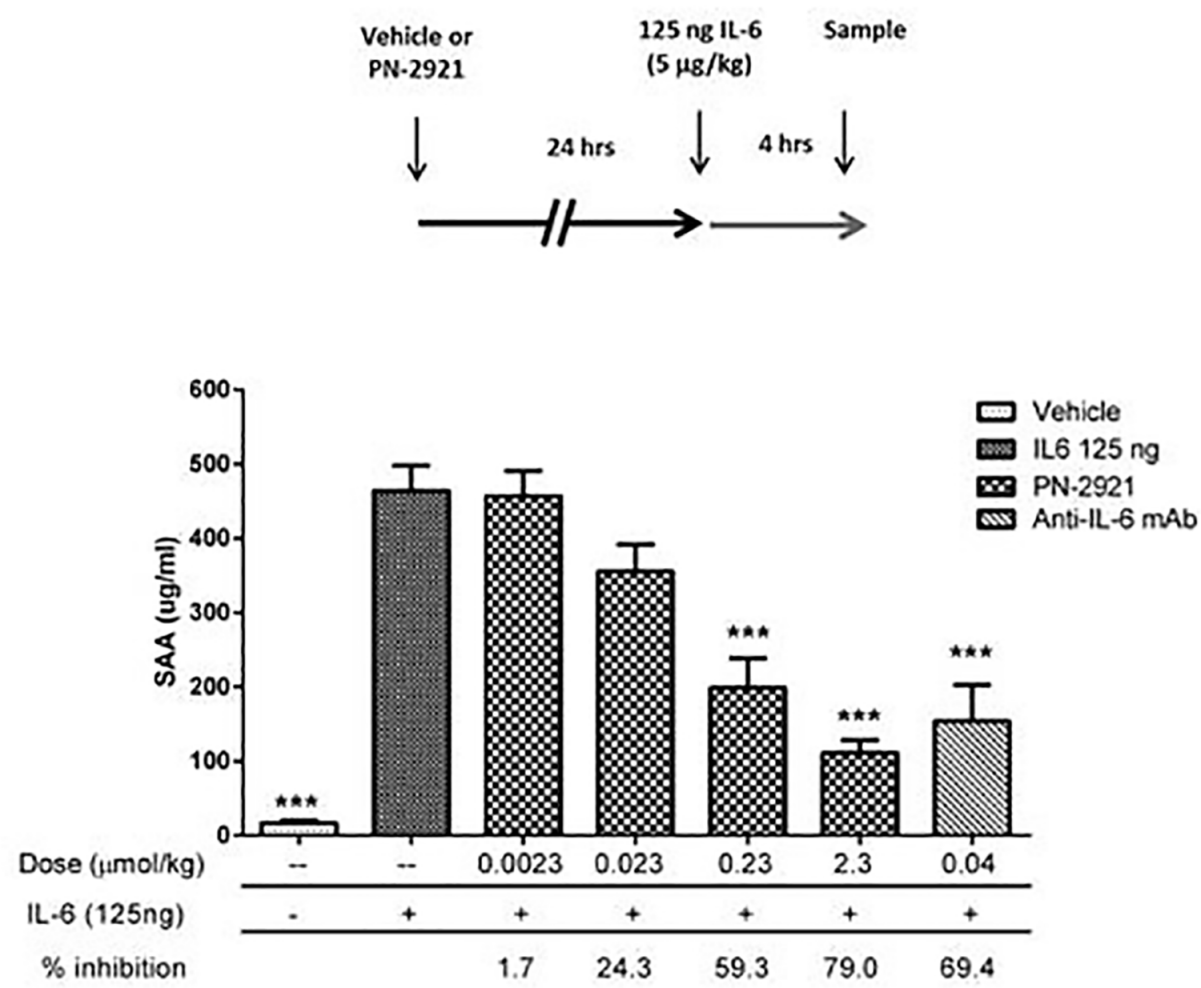
PN-2921 decreases hIL-6-induced SAA levels in mice. Balb/c mice were injected with either vehicle, the indicated dose of PN-2921, or a control anti-hIL-6 mAb. After 24 h, mice were challenged with recombinant human IL-6. After an additional 4 h, mice were sacrificed and whole blood was collected. SAA levels were determined from serum by using the mouse SAA ELISA. Statistical analysis was performed using 2-way ANOVA. *** indicates p<0.001.

PN-2921 exhibited long plasma half-lives of 23, 36 and 59 h in mice, rats and cynomolgus monkeys, respectively, after IV dosing (Fig 4 and Table 4) with low clearance values of 0.02 to 0.05 mL/min/kg. The bioavailability of PN-2921 after SC dosing was relatively high in mice and monkeys (62% and 79% respectively) and lower in rats (26%). The reason for the lower bioavailability in rats was not clear. The ability of PN-2921 to inhibit IL-6-induced biomarker responses in vivo was evaluated in a mechanistic model in which human IL-6 was acutely administered to mice to elicit SAA production. Human IL-6 was used due to the weak potency of PN-2921 against mouse IL-6 as determined by our pSTAT3 experiments in Balb/c-3T3 cells. Based on its plasma Tmax value, PN-2921 was administered SC 24 h prior to IP administration of IL-6. PN-2921 dose-dependently inhibited IL-6-induced SAA levels (Fig 5), with a calculated ED_50_ of 0.07 μmol/kg (S1 Fig).

**Table 4:** Single Dose PK parameters of PN-2921 in Mice, Rats, and Monkeys.

Species	Dose	t_1/2_	T_max_	T_last_	C_max_	AUC_0-inf_	Cl	V_ss_	F
	μmol/kg	hr	hr	hr	nM	hr*nM	mL/min/kg	mL/kg	%
Mouse	0.23	23	24	168	482	24187	0.05	90.8	62
Rat	0.23	36	36	168	299	29043	0.02	50.2	26
Monkey	0.1	59	40	336	538	66960	0.02	94	79

Summary of the PK parameters of the PN-2921 single dose PK data in mice, rat and monkeys shown in Fig 4. Both mouse and rats were dosed at 0.23 μmol/kg, while monkeys were dosed at 0.1 μmol/kg. Mouse and rat data were normalized to 0.1 μmol/kg for the purpose of comparison to monkey data.

**Fig 6:**
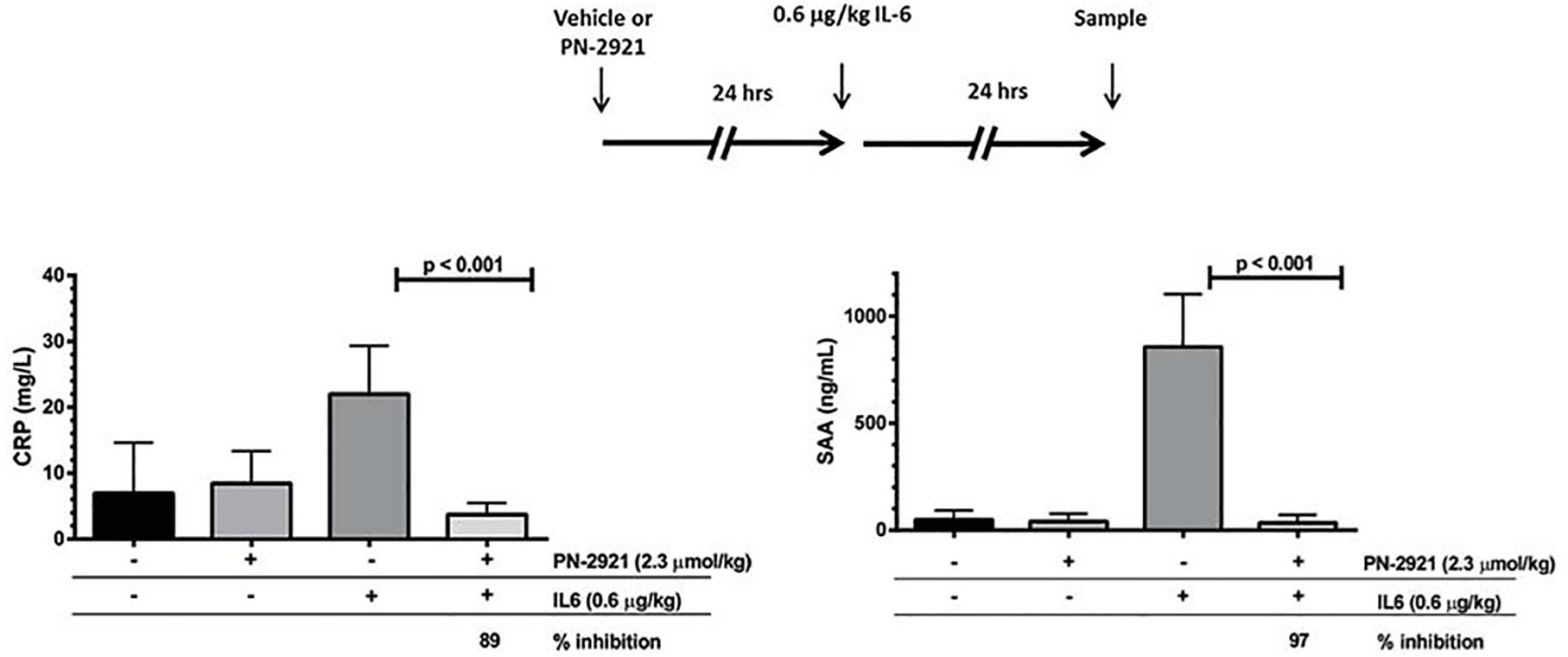
PN-2921 inhibits IL-6-induced CRP and SAA levels in cynomolgus monkey. Cynomolgus monkeys were treated SC with vehicle, and a second group of six male cynomolgus monkeys were treated SC with 2.3 μmol/kg of PN-2921 at 24 h prior to SC administration of 0.6 μg/kg IL-6. Blood samples were collected immediately prior to IL-6 administration and at 24 h post IL-6. Resultant plasma samples were assayed for CRP and SAA levels by ELISA, and plasma PN-2921 levels by LC-MS/MS. Statistical analysis was performed using 2-way ANOVA.

Based on the ability of PN-2921 to inhibit IL-6-induced biomarkers in mice, we extended these results to a higher order mammalian species. Administration of PN-2921 (2.3 μmol/kg) to cynomolgus monkey 24 h prior to an IL-6 challenge produced a significant inhibition of IL-6-induced biomarker response with 89% and 97% reduction of CRP and SAA respectively (Fig 6). Based on these studies, PN-2921 markedly suppressed IL-6-elicited biomarker response in both mice and monkeys following administration of exogenous human IL-6. Further characterization of PN-2921 in animal models of pathophysiology would require use of non-human primates or genetically modified mouse models where human IL-6 was inserted into the murine IL-6 locus.

## Conclusion

Starting from a functionally inert scaffold, a potent IL-6 binding peptide, PN-2921, was developed by a combination of molecular modeling, medicinal chemistry and phage display. PEGylation of the peptide did not impact its in vitro potency, but did result in a molecule with both a prolonged PK profile across multiple species and a potent in vivo pharmacodynamic effect, as determined by blockade of IL-6-induced acute phase responses in both mice and monkeys. To our knowledge, this is the first successful demonstration of a potent IL-6 binding peptide with prolonged PK and in vivo IL-6 antagonism, and represents a novel peptide-based approach for inhibiting the biological actions of this cytokine.

## Supporting Information

S1 Fig Dose-response relationship of PN-2921 inhibition of IL-6-induced serum SAA levels. Semi-log graph of PN-2921 dose-response data from Fig 5. The calculated ED_50_ of PN-2921 is 0.072 μmole/kg.
(TIF)

S1 Table Complete sequences of peptides shown in Table 1. The sequence of PN-2171 is also shown and is N-terminally acetylated and C-terminally α-amidated. Ogl = octylglycine.
(TIF)

S2 Table Complete sequences of peptides evaluated in Fig 2C and 2D. These four peptides differ only at position 29. PEG30L = 30 kDa linear PEG; PEG20Br = 20 kDa branched PEG (2 x 10 kDa PEG moieties); PEG40Br = 40 kDa branched PEG (2 x 20 kDa PEG moieties). Each of these peptides is N-terminally acetylated.
(TIF)

S3 Table Single Dose PK parameters of PN-2365, PN-2567, PN-2566 and PN-2520 in rats. Summary of the PK parameters of the single dose PK data of the non-PEGylated peptide PN-2365, PN-2520 (40 kDa PEG), PN-2566 (30 kDa PEG) and PN-2567 (20 kDa PEG) shown in Fig 2.
(TIF)
