# Supplementary figures and images for: Discovery and Characterization of a Potent Interleukin-6 Binding Peptide with Neutralizing Activity In Vivo
Source: PLoS One. 2015 Nov 10;10(11):e0141330. doi: 10.1371/journal.pone.0141330 (PMC4640888; doi:10.1371/journal.pone.0141330)

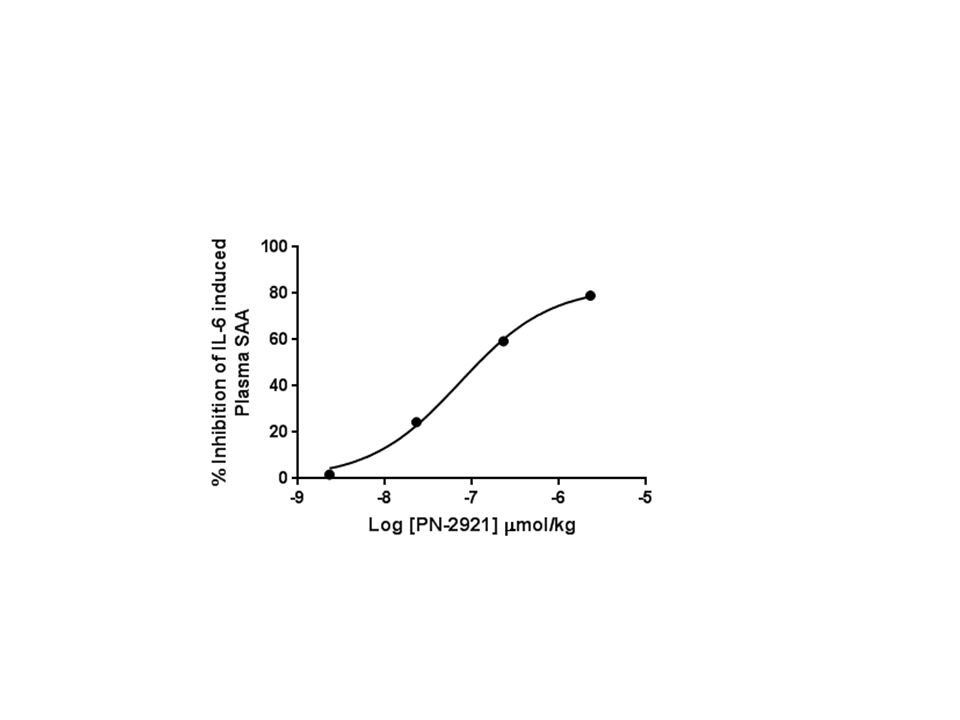

Supplement: S1 Fig — Semi-log graph of PN-2921 dose-response data from Fig 5. The calculated ED50 of PN-2921 is 0.072 μmole/kg. (TIF) [file pone.0141330.s001.tif]

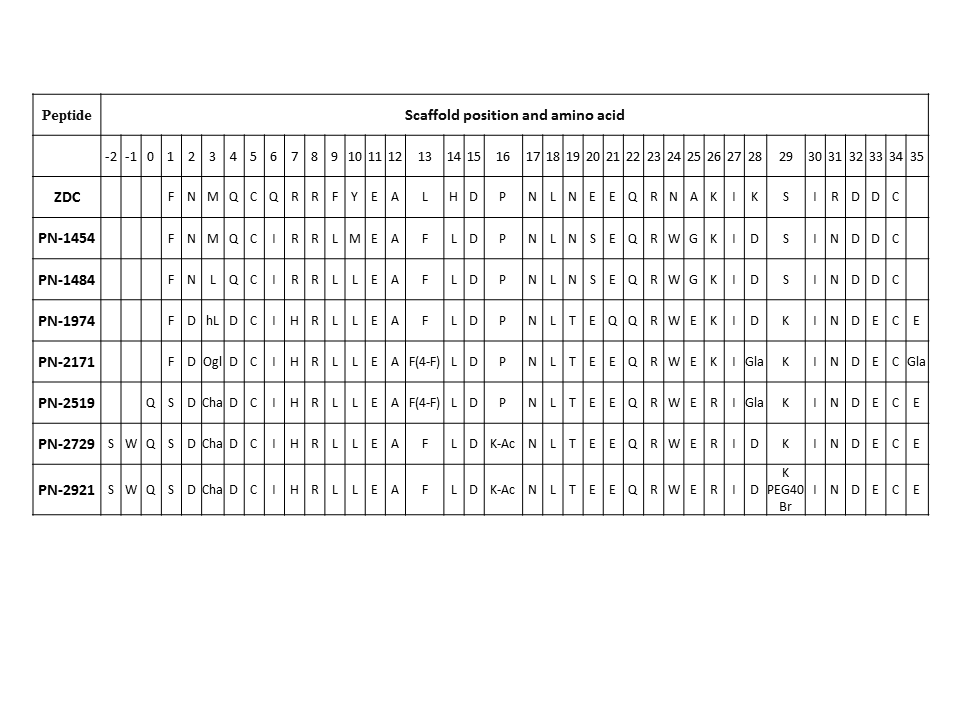

Supplement: S1 Table — The sequence of PN-2171 is also shown and is N-terminally acetylated and C-terminally α-amidated. Ogl = octylglycine. (TIF) [file pone.0141330.s002.tif]

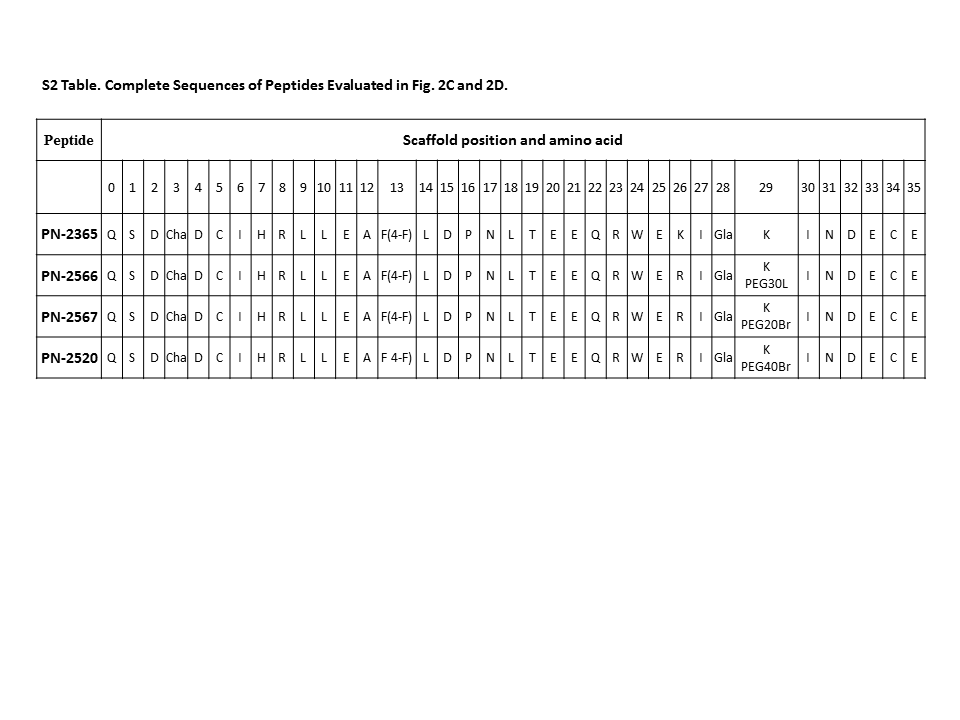

Supplement: S2 Table — These four peptides differ only at position 29. PEG30L = 30 kDa linear PEG; PEG20Br = 20 kDa branched PEG (2 x 10 kDa PEG moieties); PEG40Br = 40 kDa branched PEG (2 x 20 kDa PEG moieties). Each of these peptides is N-terminally acetylated. (TIF) [file pone.0141330.s003.tif]

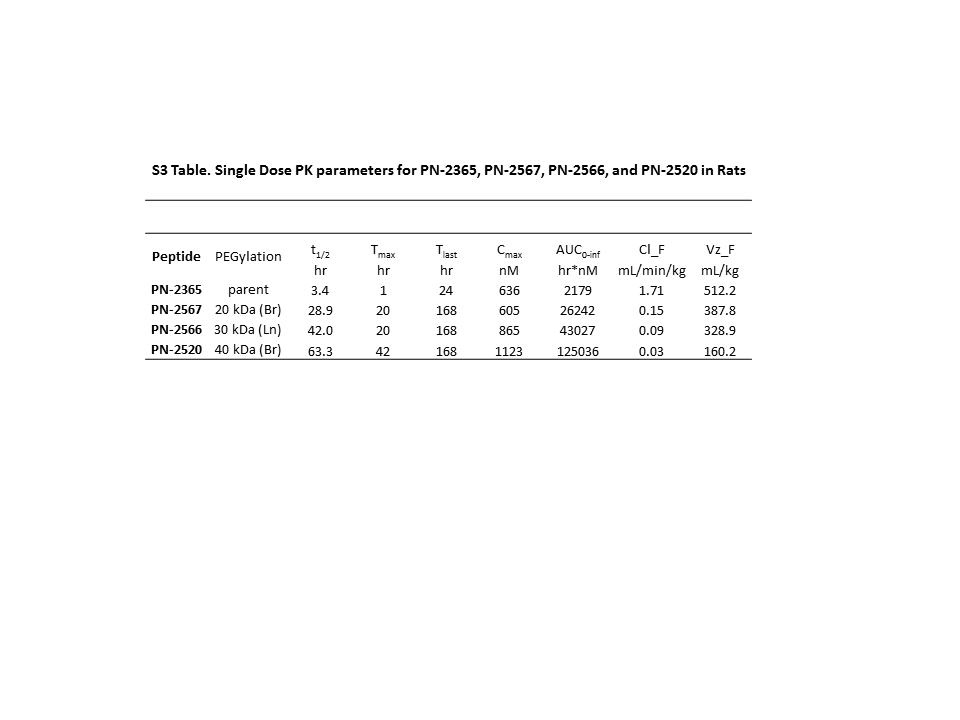

Supplement: S3 Table — Summary of the PK parameters of the single dose PK data of the non-PEGylated peptide PN-2365, PN-2520 (40 kDa PEG), PN-2566 (30 kDa PEG) and PN-2567 (20 kDa PEG) shown in Fig 2. (TIF) [file pone.0141330.s004.tif]
